# Using ConTemplate and the PDB to explore conformational space: on the detection of rare protein conformations

**DOI:** 10.1186/1471-2105-16-S3-A3

**Published:** 2015-02-13

**Authors:** Aya Narunsky, Haim Ashkenazy, Rachel Kolodny, Nir Ben-Tal

**Affiliations:** 1 Department of Biochemistry and Molecular Biochemistry, George S. Wise Faculty of Life Sciences, Tel Aviv University, Ramat Aviv 69978, Israel; 2 The Department of Cell Research and Immunology, George S. Wise Faculty of Life Sciences, Tel Aviv University, Ramat Aviv 69978, Israel; 3 Department of Computer Science, University of Haifa, Mount Carmel, Haifa 31905, Israel

## Background

Conformational changes mediate important protein functions, such as opening and closing of channel gates, activation and inactivation of enzymes, etc. The entire conformational repertoire of a given query protein may not be known; however, it may be possible to infer unknown conformations from other proteins. We developed the ConTemplate method to exploit the richness of the Protein Data Bank (PDB)[1] for this purpose. ConTemplate uses a three-step process to suggest alternative conformations for a query protein with one known conformation [2]. First, ConTemplate uses GESAMT to scan the PDB for proteins that share structural similarity with the query [3]. Next, for each of the collected proteins, additional known conformations are detected using BLAST [4], and clustered into a predefined number of clusters [5]. Finally, MODELLER [6] builds models of the query in various conformations, each representative of a cluster.

## Results

**Figure 1:**
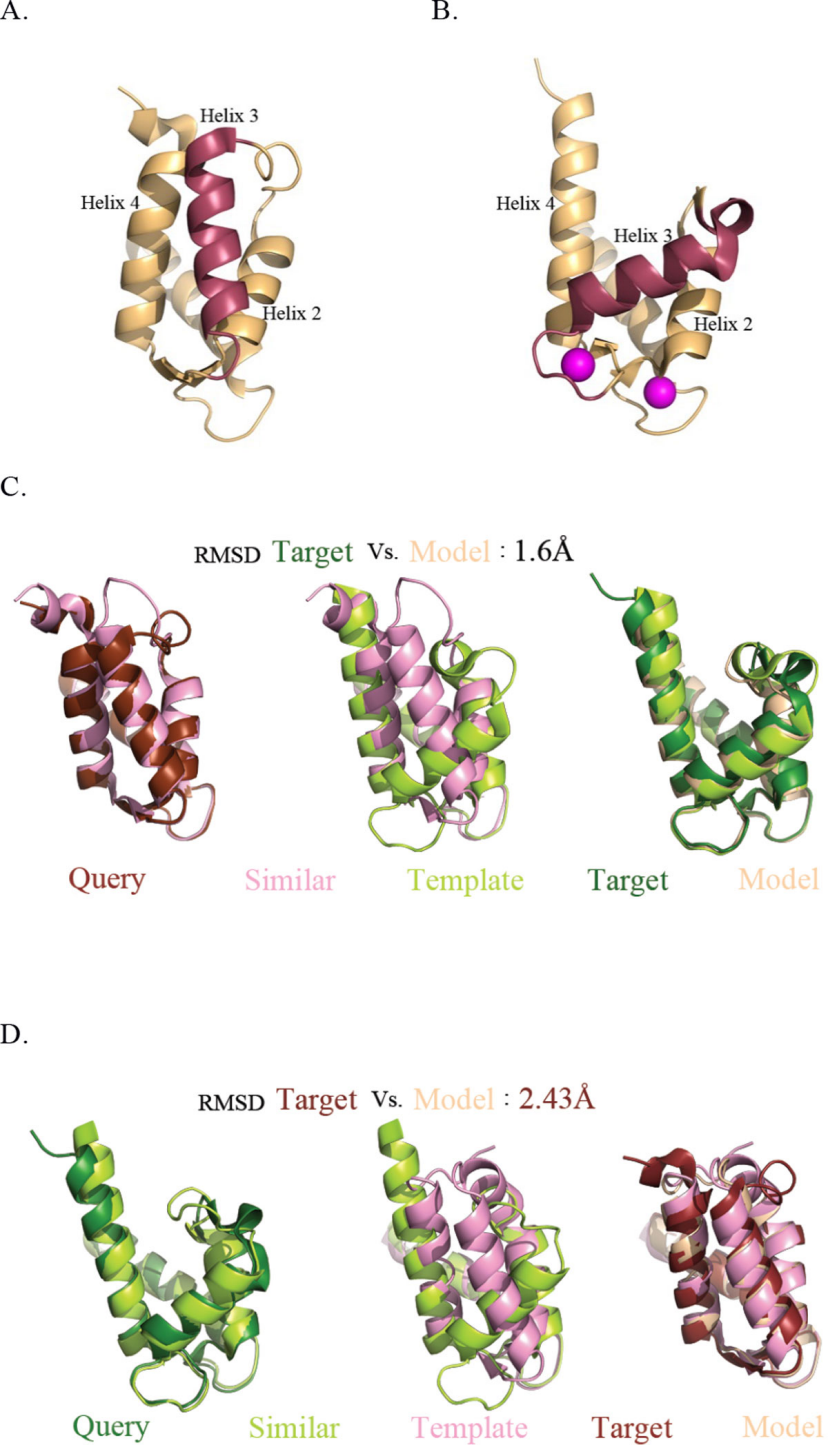
**ConTemplate results demonstrated using the S100A6 Ca^2+ ^binding protein**. The Ca^2+^-free (**A**) and **-**bound (**B**) conformations are shown in the upper panels; helix 3 is marked in red, and the calcium ions in magenta. **C**. Reproducing the Ca^2+^-bound conformation, starting from the Ca^2+^-free conformation as a query. The maximal RMSD between the query and similar proteins is set to 1.2Å, the minimal Q-score to 0.4, and the number of clusters is set to 2. **D**. Reproducing the Ca^2+^-free conformation, starting from the Ca^2+^-bound conformation as a query. The similarity cutoffs are the same as in **C**, the number of clusters is set to 17.

We demonstrate the application of ConTemplate with S100A6, a member of the S100 family of Ca^2+ ^binding proteins. The vast majority of proteins in this family bind Ca^2+ ^through helix-loop-helix EF-hand motifs. The structure of the protein includes four helices connected by three loops. Calcium binding is coupled to a conformational change, in which helix 3 changes its orientation with respect to helix 4 (Figure 1A and 1B) [7]. Helix 2 also changes its positioning with respect to the rest of the protein upon calcium binding, but the change is not as dramatic. The RMSD between the Ca^2+^-bound and -free conformations is 4.46Å. The EF-hand motif is found in many PDB entries. Yet, known structures of the Ca^2+^-free conformation are relatively rare. These features make the protein an interesting example for examining how the performance of ConTemplate is affected by the distribution of conformations in the PDB: The highly abundant Ca^2+^-bound conformation may populate a very large cluster, which could mask the Ca^2+^-free conformation. Thus, finding the latter conformation could be challenging.

Starting from the Ca^2+^-free conformation as a query, it is sufficient to set the number of clusters at 2 to retrieve both the Ca^2+^-bound and -free conformations. ConTemplate reproduces the Ca^2+^-bound conformation with RMSD of 1.6Å (Figure 1C). This is based on the query's structural similarity to the Ca^2+^-free conformation of another member of the family, the S100A2 protein [8], and the bound conformation of this protein [9]. The sequence identity between the two proteins is 47%. When the number of clusters is set to be larger than 2, each cluster represents either the Ca^2+^-bound or the Ca^2+^-free conformation. On the other hand, using the abundant Ca^2+^-bound conformation as a query, even with up to three clusters, the process retrieves only variants of the (initial) bound conformation. Only when the number of clusters is four or larger do we obtain at least one cluster representing the Ca^2+^-free conformation. In general, the ability to predict the other conformation improves as the number of clusters increases. For example, with 17 clusters, 4 clusters represent the rare conformation, and ConTemplate reproduces the Ca^2+^-free conformation with RMSD of 2.43Å (Figure 1D). This is based on the query's structural similarity to the bound conformation of another member of the family, the S100A12 protein [10], and the known free conformation of this protein [11]. The sequence identity between the query and the template is 42%.

## Conclusions

ConTemplate suggests putative conformations for a query protein with at least one known structure, based on the query's structural similarity to other proteins. In principle, the clustering method enables the detection of distinct conformations, including local conformational changes. However, it may be necessary to adjust ConTemplate's parameters to reveal such changes, especially when looking for rare conformations. When ConTemplate suggests models that are similar to the query, and the clusters are very large, this may indicate that less-common conformations of the query are masked by highly-abundant conformations. Increasing the number of clusters may enable the rarer conformations to be detected. When the additional conformation is not known, it is not trivial to detect the "correct" conformation among the suggested models. A careful examination of the similar proteins and their conformational changes can be useful towards selecting the most probable conformations for the query. In addition, if the number of clusters is large enough, a pathway between the query conformation and a putative conformation may be found, with other models serving as intermediates. Identification of such a pathway could provide insight into the physiological relevance of a newly-detected conformation.
